# The immunopathology of canine vector-borne diseases

**DOI:** 10.1186/1756-3305-4-48

**Published:** 2011-04-13

**Authors:** Michael J Day

**Affiliations:** 1 School of Veterinary Sciences, University of Bristol, Langford BS40 5DU, UK

## Abstract

The canine vector-borne infectious diseases (CVBDs) are an emerging problem in veterinary medicine and the zoonotic potential of many of these agents is a significant consideration for human health. The successful diagnosis, treatment and prevention of these infections is dependent upon firm understanding of the underlying immunopathology of the diseases in which there are unique tripartite interactions between the microorganism, the vector and the host immune system. Although significant advances have been made in the areas of molecular speciation and the epidemiology of these infections and their vectors, basic knowledge of the pathology and immunology of the diseases has lagged behind. This review summarizes recent studies of the pathology and host immune response in the major CVBDs (leishmaniosis, babesiosis, ehrlichiosis, hepatozoonosis, anaplasmosis, bartonellosis and borreliosis). The ultimate application of such immunological investigation is the development of effective vaccines. The current commercially available vaccines for canine leishmaniosis, babesiosis and borreliosis are reviewed.

## Introduction

Vector-borne diseases affecting the domestic dog are of major global significance for their impact on the health and well being of these companion and working animals, and also because for some of these diseases the dog acts as a reservoir species for infection of the human population. The most significant of these diseases are bacterial and microparasitic and these are summarized in Table 1. These diseases have received the attention of the veterinary and public health research communities in recent years and progress in such research forms the focus of the annual series of Canine Vector-Borne Disease (CVBD) workshops hosted by Bayer and now summarized in the pages of this journal.

**Table 1:** Major canine vector-borne diseases

Infectious Agent	Arthropod Vectors	Zoonotic Potential	Reference
*Leishmania infantum*	*Phlebotomus *sandflies (old world)	Dog is major reservoir of infection	[17]
*(Leishmania chagasi)*	*Lutzomyia *sandflies (new world)		

*Babesia vogeli*	*Rhipicephalus sanguineus*	Not with canine pathogens	[103]
*Babesia canis*	*Dermacentor *spp.		
*Babesia rossi*	*Haemaphysalis leachi*		
Other large *Babesia*			
*Babesia gibsoni*	*Haemaphysalis *spp. *Rhipicephalus sanguineus?*		
*Babesia conradae*	Unknown		
*Babesia microti*-like (also known as *Theileria annae*)	*Ixodes hexagonus *(suspected)		

*Hepatozoon canis *	*Rhipicephalus sanguineus*	Unlikely due to mode of transmission (ingestion of vector)	[104]
*Hepatozoon americanum*	*Amblyomma maculatum*		

*Ehrlichia canis*	*Rhipicephalus sanguineus*	*E. ewingii and E. chaffeensis *are human pathogens, but role of the dog as a reservoir is unproven; human infections with *E. canis *are reported	[105,106]
*Ehrlichia ewingii*	*Amblyomma americanum*		
*Ehrlichia chaffeensis*	*Amblyomma americanum*		

*Anaplasma phagocytophilum*	*Ixodes ricinus*	Important human pathogen	[105-107]

*Anaplasma platys*	*Rhipicephalus sanguineus *(suspected)	None recognized unequivocally	[105,106]

*Rickettsia rickettsii *(Americas)	*Dermacentor andersoni*	Important human pathogen; people may become infected whilst removing engorged ticks from dogs; dogs maintain infested tick population in the domestic environment	[105]
	*Dermacentor variablis*		
*Rickettsia conorii *(Europe, Asia, Africa)	*Rhipicephalus sanguineus*		

*Borrelia *(multiple species but primarily *B. burgdorferi sensu stricto, B. garinii *and *B. afzelii*)	*Ixodes *ticks (multiple species)	Dog is an 'accidental host' but may carry ticks into the domestic environment	[108,109]

*Bartonella vinsonii *subspecies *berkhoffii*	Ticks proposed (fleas for cats)	Unknown if dogs are competent reservoirs; *B. vinsonii *subsp. *berkhoffii *(predominant canine isolate) is a rare cause of human infections	[36,40]
*Bartonella henselae*			
*Bartonella clarridgeiae*			
*Bartonella rochalimae*			
*Bartonella quintana*			
*Bartonella washoensis*			

*Dirofilaria immitis*	Mosquitoes	Rare human infections; incidental host	[110]

*Mycoplasma haemocanis *Candidatus *Mycoplasma haematoparvum*	*Rhipicephalus sanguineus *(proposed)	No evidence for human infection	[111]

The greatest research activity has focussed on the molecular speciation of the infectious agents, definition of their parasite vectors, the geographical distribution and movement of agents and vectors, the clinical syndromes expressed by infected dogs and people and elements of the pathogenicity of the causative organisms. The more challenging aspect for research remains an exploration of the pathology and secondary immunopathology established in dogs by these infections and the nature of the immune response made by the canine host to the pathogens. There are practical reasons why this important area of research has been poorly addressed to date, including: (1) the ethics and expense of working with the dog as an experimental animal, (2) the problem, for some diseases, of establishing reliable and repeatable in-vivo model systems, (3) the challenges in assembling significantly large populations of spontaneously-infected and well-characterized clinical populations, (4) the availability of appropriate immunological reagents and molecular methodology for dissection of the canine immune response relative to such investigations in man or laboratory rodents, and (5) the difficulty in attracting appropriate research funding for the investigation of canine disease. With the publication of versions of the canine genome [1] there have been rapid advances with respect to investigative technology for canine immunopathological research, so this is now less of an insurmountable problem. Numerous studies have shown the utility of the reverse transcriptase polymerase chain reaction (RT-PCR) for exploring the tissue expression of targeted genes [2], although the application of methods such as mRNA expression microarrays [3] and genome wide association studies (GWAS) [4,5] remain costly options.

The aim of the present review is to summarize the current state of knowledge regarding the pathology and immunology of CVBD in the host species. The review will focus on key diseases and cannot be exhaustive, but should give a feel for the current state-of-the-art in this important area of veterinary medicine. It should be noted that the scope of the review does not include the equivalent feline infections and that for at least one of these (feline bartonellosis; [6]) knowledge of immunopathology has progressed beyond that currently described for the dog.

## Unique aspects of the canine vector-borne diseases

**Figure 1:**
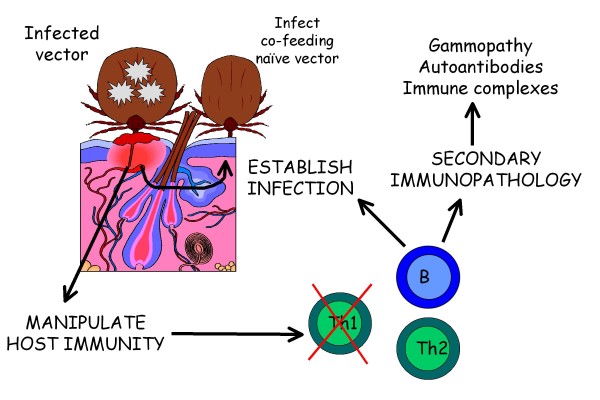
**The triad of canine vector-borne disease**. The canine vector-borne diseases are characterized by the unique three-way interaction between the infectious agent, the vector and the host immune system. Vector salivary proteins injected into the dermal microenvironment during taking a blood meal modulate the host immune system creating a favourable environment for survival and replication of the infectious agent. This permits infection of co-feeding naïve vectors. The effects on host immunity are often to promote Th2-regulated humoral responses above the protective Th1-regulated cellular immune response. This allows persistence of the infection and encourages the development of inappropriate secondary immunopathology characterized by hypergammaglobulinaemia, autoantibody and immune complex formation.

In addition to the practical issues defined above, simple aspects of the nature of the CVBDs make them particularly challenging for investigation. All of these infections are characterized by being formed of a unique triad that involves the infectious agent, the vector of that agent and the host animal (Figure 1) [7,8]. In many instances this integral and complex three-way relationship has likely evolved over millennia with fascinating selective advantages conferred by some aspects of the relationships. For example, the infectious agents are known to be able to manipulate the physiology (gene expression) and behaviour of the arthropod vectors to ensure their transmission [9] and during the process of taking a blood meal the arthropod vectors appear to be able to manipulate the host immune system through the release of potent salivary immunomodulators, thereby conferring an advantage to the co-transmitted microorganism in terms of establishment of disease and further transmission to naïve co-feeding vectors via the process of 'saliva-activated transmission' [8,10]. This group of infectious agents is also characterized by the ability to induce chronic or recrudescent disease in the canine host, likely by occupying particular tissue niches where they may be protected from the host immune response, or by manipulating host immunity to prevent a sterilizing response and permit persistent infection. The clinicopathological changes in any one dog may be complicated by the co-infections that may occur in endemic areas (e.g. combinations of leishmaniosis, monocytic ehrlichiosis and babesiosis; or borreliosis and anaplasmosis). Finally, the nature of the clinical signs expressed by the infected dog often relate to secondary immunopathology (e.g. the induction of autoantibodies or formation and deposition of immune complexes of antigen, antibody and complement) rather than direct cellular or tissue damage and the accompanying inflammatory response. With all of these complex interrelationships, our greatest knowledge gap relates to what is happening within the infected canine host.

## The pathology of canine vector-borne diseases

The simple pathology of the CVBDs is relatively poorly described. Few investigations have ever been made of the primary site of interaction of the agent-vector-host triad, i.e. the cutaneous site of vector attachment and feeding, which also represents the site of transmission of the agent and the point of initial engagement with the host immune system. The histopathology of canine cutaneous tick-attachment sites has been described at the light microscopical level, including the central dermal cone-shaped zone of necrosis related to insertion of tick mouthparts and formation of 'cement' and the surrounding mixed chronic inflammatory infiltration of macrophages, lymphocytes, plasma cells, neutrophils and eosinophils [8,11]. However, given the increasing range of species-specific and cross-reactive reagents now available for immunohistochemical evaluation of canine tissue immune responses [12] it is surprising that the temporal kinetics and nature of these inflammatory responses has not been further defined. Such studies have been performed recently in sheep exposed twice to *Hyalomma *ticks, in which cutaneous attachment sites and regional draining lymph nodes had infiltration of CD1^+ ^dendritic cells, CD8^+ ^cytotoxic T lymphocytes and T cells bearing the γδ form of the T-cell receptor [13].

**Figure 2:**
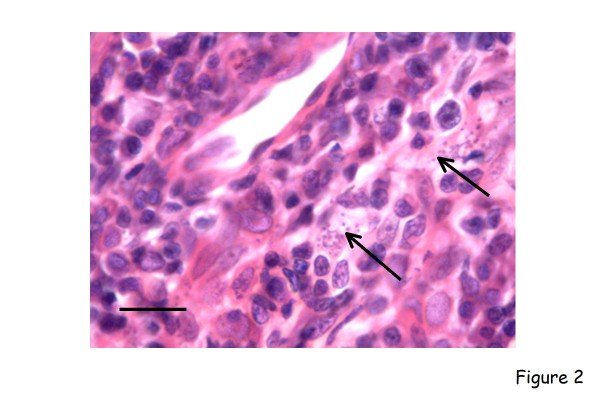
**Canine leishmaniosis**. Skin biopsy from a dog with symptomatic visceral leishmaniosis. There is a mixed chronic inflammatory infiltration of the dermis associated with numerous macrophages laden with amastigotes (arrows). Haematoxylin and eosin, bar = 50 μm.

There have been numerous reports of the cutaneous pathology in the chronic stages of canine leishmaniosis (Figure 2) and in some of these the presence of intracellular amastigotes within the macrophages forming the granulomatous dermatitis has been highlighted immunohistochemically [14,15]. Immunohistochemical studies have also shown that in the relatively milder clinical lesions of exfoliative dermatitis there is a low parasite burden, associated with enhanced expression of class II molecules of the major histocompatibility complex (MHC) by keratinocytes and a dermal T-cell infiltrate dominated by CD8^+ ^cells over the CD4^+ ^T helper (Th) subpopulation. In contrast, with increasing severity of cutaneous disease (the nodular form) there is reduced expression of class II molecules of the MHC by epidermal Langerhans cells and keratinocytes and fewer infiltrating T lymphocytes [16,17]. The lymph nodes of asymptomatic *Leishmania*-infected dogs are hyperplastic, but when disease becomes symptomatic there is more often atrophy of the lymph node cortex [18].

**Figure 3:**
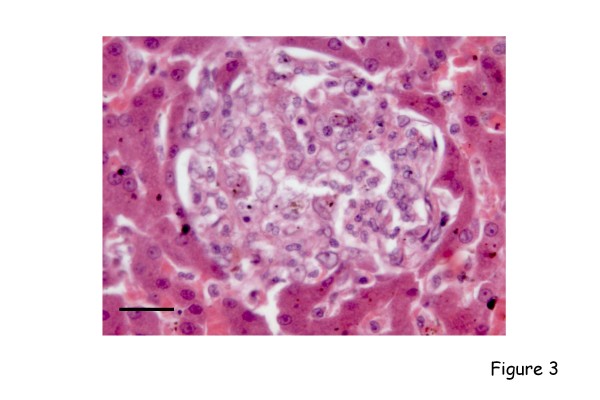
**Canine leishmaniosis**. Section of liver from a dog with visceral leishmaniosis. There is a discrete focus of granulomatous inflammation within the mid-zonal hepatic parenchyma. Macrophages within the focus will contain amastigotes. Haematoxylin and eosin, bar = 50 μm.

**Figure 4:**
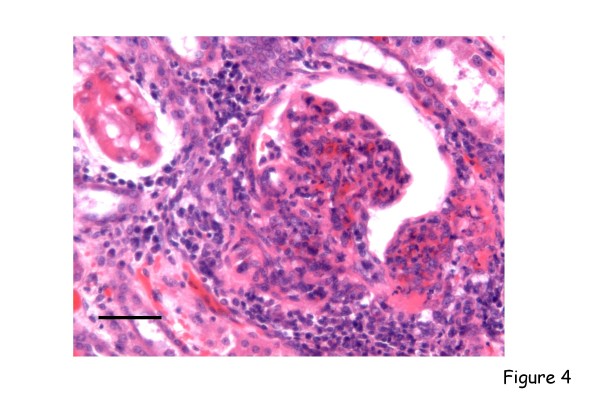
**Canine leishmaniosis**. Section of kidney from a dog with visceral leishmaniosis. There is marked lymphoplasmacytic interstitial nephritis with obliteration of the glomerulus and afferent and efferent arterioles by granulomatous inflammatory infiltration. Haematoxylin and eosin, bar = 100 μm.

Limited investigations have been performed on the systemic pathological changes that accompany these diseases in spontaneously- or experimentally-infected dogs. Again, the most reported are the lesions that develop in canine leishmaniosis [19], for example the granulomatous inflammatory infiltrates that form with foci of infection in organs such as the liver [20] (Figure 3), and the range of secondary immunopathological (e.g. immune complex-mediated) lesions that arise in the renal glomeruli [21-23] (Figure 4), the nasal mucosa [24] or the uveal tract of the eye [25,26]. Immunohistochemistry has been used in several studies to determine the parasite load of infected tissues [27].

Pathological descriptions have also been made of the synovial, renal ('Lyme nephropathy') and nervous system lesions in canine borreliosis [28,29]. The renal lesions are characterized by glomerulonephritis (with immunoglobulin [Ig] G, IgM and complement C3 subendothelial immune complex deposition), interstitial nephritis and tubular necrosis [29]. A detailed histopathological description of the lesions in dogs infected experimentally by exposure to infected ticks has been reported. These animals developed mixed inflammatory dermatitis at the site of tick attachment, hyperplasia within the draining lymph node and synovitis of nearby forelimb joints. The synovial reaction was either predominantly an acute neutrophilic inflammation with intraarticular fibrin deposition or a chronic lymphoplasmacytic reaction with plasma cells dominating over CD3^+ ^T cells. Perineuritis and periarteritis were also described in the periarticular tissue [30]. In another experimental infection study, lymphoplasmacytic meningitis was also recorded in 3/20 infected dogs [31].

The bone marrow changes in chronic monocytic ehrlichiosis have been reported [32]. Dogs infected experimentally with *Ehrlichia canis *develop lymphoplasmacytic uveitis and meningitis [33] and experimental infection with *Anaplasma phagocytophilum *leads to splenic hyperplasia and mild non-specific reactive hepatitis [34]. The spectrum of *Bartonella*-associated inflammatory changes in individual canine patients, particularly dogs with endocarditis [35-40], has been described. The pathology of lesions of skeletal muscle [41] and the periosteum [42] has been described in dogs infected by *Hepatozoon americanum*. These are all primarily light microscopical descriptions and the lesions await further exploration by immunohistochemistry or characterization of lesional gene expression via RT-PCR or microarray investigation of fresh-frozen tissue extracts.

## The immunology of canine vector-borne diseases

**Figure 5:**
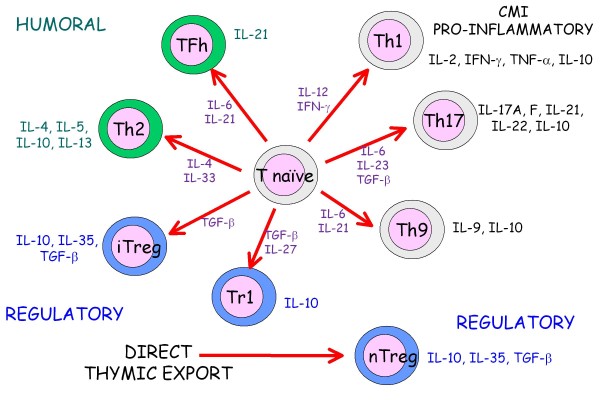
**CD4^+ ^T-cell subsets**. There is increasing complexity in the network of functional CD4^+ ^T-cell subsets. Under the influence of specific promoting cytokines (purple lettering) and transcription factors (not shown here), the naïve T cell may differentiate towards a functional subset promoting either cell-mediated and proinflammatory immunity (Th1, Th17 and Th9; black lettering), humoral immunity (Th2 and T follicular helper [TFh]; green lettering) or a suppressive response (induced Treg and Treg1; blue lettering). These subsets are not strongly polarized and there is considerable 'plasticity' in their actions, whereby one cell type can be reprogrammed to another at a different stage of the immune response. This is readily seen by the ability of multiple of the subsets to produce the immunosuppressive cytokine IL-10. The natural Treg appear separately, as this committed lineage leaves the thymus directly and is responsible for the control of allergen- and autoantigen-specific lymphocytes.

**Figure 6:**
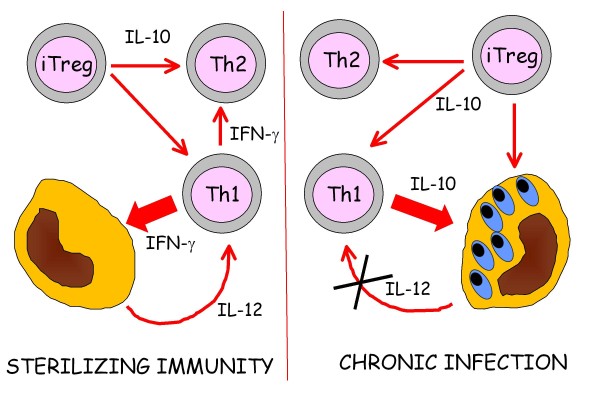
**Plasticity of CD4^+ ^T-cell subsets**. The plasticity of CD4^+ ^T-cell subsets is demonstrated in the murine model of leishmaniosis. In many chronic infectious diseases it is now recognized that sterilizing immunity is prevented by the action of T cells with regulatory function. Although regulatory T cells prevent complete elimination of the infection, they are crucial in inhibiting the development of secondary immunopathology. A balance is therefore achieved between infection-limiting Th1 immunity and immunopathology-limiting regulation. The regulatory activity might come from classical induced or natural Treg, but equally some *Leishmania*-specific Th1 cells may be re-programmed to become IL-10 producing regulatory cells.

Understanding of the immune response to infection has expanded greatly in recent years and, as for many aspects of immunology, this new knowledge is based on application of the discovery of multiple functional subsets of T lymphocytes expressing the CD4 co-receptor molecule. The diversity in regulatory T-cell subsets (Figure 5) is best characterized in experimental rodents and man. Following the initial description of Th1 and Th2 cells by Mossman et al. [43] much of the early work defining the interrelationships between these populations was performed using the murine model of dermatotropic leishmaniosis. In this model there was clear dichotomy between the protective Th1 immune response in disease-resistant C57Bl6 mice and the Th2 response made by susceptible BALB/c mice [44]. More recently, the murine model system has enabled definition of the role of regulatory T lymphocytes (Treg) in chronic infections such as leishmaniosis. These cells inhibit the function of Th1 cells allowing persistence of infection, but more importantly limit the development of secondary immunopathology. In murine models it has also been suggested that effector Th1 cells may become a source of the key regulatory cytokine, interleukin (IL)-10, for the same reason [45] (Figure 6).

The CD4 Th subset paradigm has also been used as a framework for understanding the immunomodulatory properties of arthropod salivary proteins released into the host dermal microenvironment upon taking a blood meal. Arthropod saliva is not only anticoagulant and anti-inflammatory in nature, but also contains potent immunomodulators that are now being characterized and purified. In general terms, the effect of these salivary molecules is to alter the host immune response within the skin and possibly also within draining lymphoid tissue. These molecules tend to promote Th2 immunity while suppressing the Th1 response. This provides a more favourable environment for the establishment of intracellular infection (for which Th1 immunity is the appropriate means of clearance of infection) and at the same time pushes immune balance towards conditions favouring secondary humoral immunopathology (e.g. autoantibody and immune complex formation) [8]. Few studies have examined the effect of arthropod salivary proteins on the canine immune system. Salivary gland extract from *Rhipicephalus sanguineus *co-cultured with canine peripheral blood mononuclear cells suppressed the production of total immunoglobulin and IgA (but not IgM) by lymphocytes stimulated with pokeweed mitogen or lipopolysaccharide [46].

For leishmaniosis, clear parallels were recognized between the susceptibility and resistance phenotypes in murine strains and in natural infection of the dog. Relatively early studies confirmed that the resistance of certain dogs to severe clinical leishmaniosis was also determined by a Th1 immune response with production of the key Th1 cytokine interferon (IFN)-γ [47]. Leishmaniosis has since become the single best understood vector-borne disease of the dog as it has rightly attracted appropriate funding commensurate with the significance of the canine disease for human health. Numerous investigations have confirmed the importance of Th1 immunity and further characterized cytokine gene expression in this disease. Asymptomatic spontaneously-infected dogs have greater expression of genes encoding IFN-γ and tumour necrosis factor (TNF)-α and a lower parasite burden within lymph nodes, compared with symptomatic dogs with high parasite burdens in which there is greater expression of genes encoding the immunosuppressive cytokines IL-10 and transforming growth factor (TGF)-β [48]. In contrast, within the spleen there was no clear difference in IFN-γ, IL-4 and IL-12 gene expression between asymptomatic and symptomatic dogs [49], but splenic IL-10 mRNA expression was greater in symptomatic dogs [50]. There was no clear evidence for higher expression of IL-10 mRNA in the bone marrow or blood lymphocytes of infected versus uninfected or symptomatic versus asymptomatic dogs [51,52]. A study of cytokine gene expression using RNA extracted from formalin-fixed tissue biopsies and real-time RT-PCR has shown elevation of mRNA encoding IL-4, IFN-γ and TNF-α compared with normal skin, but an association between cutaneous parasite burden and severity of skin lesions with only IL-4 gene expression. This was interpreted to suggest a Th2 bias within the cutaneous lesions [53].

Overall, these cytokine gene expression studies suggest that symptomatic infected dogs have insufficient Th1 (IFN-γ) with enhanced Treg (IL-10) activity, however interpretation of these investigations should be tempered by the facts that: (1) there is little standardization of the disease (e.g. spontaneous versus experimental infection, breed of dog, geographical setting, infectious load etc.), (2) there is variation in the body compartment tested (e.g. blood, lymph node, spleen, bone marrow or skin), (3) most of the studies employ conventional gel-based RT-PCR rather than real time RT-PCR employing multiple housekeeper genes for relative determination of copy number [54], and (4) gene expression rather than protein production is measured.

In the peripheral blood of symptomatic dogs there is a reported reduction in the proportions of circulating CD21^+ ^B cells and CD14^+ ^monocytes compared with asymptomatic dogs in which the major alteration is an elevation in the frequency of CD8^+ ^T cells [55]. However, these data are controversial, as other studies have shown no difference in the proportions of different blood lymphocyte subpopulations between normal and infected dogs and in infected symptomatic dogs throughout the course of medical therapy [56]. The lymph nodes of *Leishmania*-infected dogs have proportionally more CD8^+ ^T cells, reduced B cells and upregulation of MHC class II expression by lymphoid cells [18].

It has proven much more challenging to clearly relate the subisotype of the IgG antibody response to clinical status. Although there are clear associations between murine Th1 and IgG2a responses and Th2 and IgG1 responses, there is no consensus as to whether canine humoral immune responses are polarized in leishmaniosis. The confusion partly arises from inconsistency in nomenclature and the validity and specificity of reagents produced for the detection of canine IgG subclasses [57,58].

The genetic basis for protective immunity as recognized in particular breeds (e.g. the Ibizian hound and dogs raised in endemic areas) [59] has been explored by characterizing associations with genes of the canine MHC (the DLA system; specifically the DLA class II allele DLA-DRB1*01502) [60] and genes identified as associated with resistance in other species (*Slc11a1 *encoding the natural resistance associated protein 1 [NRAMP1]) [61]. A recent study has failed to confirm sequence differences in *Slc11a1 *between symptomatic and asymptomatic naturally-infected dogs [49]. The most exciting current investigation of canine leishmaniosis is that which forms one of the work packages of the European Union-funded LUPA project http://www.eurolupa.org/, in which susceptibility and resistance is being examined via GWAS studies of significant numbers of dogs with clinically well-characterized infection status and disease.

In contrast to leishmaniosis, the host immune response to the remaining major canine vector-borne pathogens is relatively poorly characterized. Logically, most of the focus for these infections has been on understanding the humoral immune response in order to develop serological diagnostic methods for detection of individual cases or population epidemiological studies. The combination of serology and PCR detection now forms the cornerstone for diagnosis in most of the CVBDs, but it is beyond the scope of this review to detail studies of the kinetics of the humoral immune response in the CVBDs.

There has been very limited exploration of other aspects of the immune response in these diseases; in particular how these infections might map to the CD4 T cell immunoregulatory model described above for leishmaniosis. Mixed breed dogs infected experimentally with *E. canis *and monitored for 4 months post infection had no significant changes in the concentration of serum IgG, IgM and IgA, the percentage of circulating CD4^+ ^T cells or the in-vitro function of blood lymphocytes (as determined by mitogen stimulation and the ability of lymphokine-activated killer cells to cause lysis of a ^51^Cr-labelled target monocyte cell line). A transient elevation in blood CD8^+ ^T cells was found at 6 weeks post infection [62]. Whilst the study of T cell and cytokine responses may not be crucial for diagnosis of the CVBDs, such knowledge should underpin the development of vaccines and immunotherapeutic approaches to the management of these infections.

In addition to the host immune response to the vector-borne agents, an important part of the pathogenesis of these infections often involves the secondary immune-mediated sequela that are manifestations of immune dysregulation induced by the organism and/or the vector [7]. Many of the CVBDs are characterized by serum hypergammaglobulinaemia and the production of circulating immune complexes or autoantibodies (e.g. antierythrocyte and antiplatelet antibodies, antinuclear antibodies) that play a role in disease pathogenesis. Immune complex deposition and autoantibody formation is widely recognized in the pathogenesis of canine leishmaniosis [63-65] and monocytic ehrlichiosis [66,67]. In the latter disease, both platelet-bound and serum platelet-bindable antibodies have been demonstrated [68,69], in addition to antinuclear antibodies and antierythrocyte antibodies [70]. Dogs with *A. phagocytophilum *infection have also been reported as being Coombs positive and having platelet-bound antibodies [71,72]. Similarly, 11/16 dogs with Rocky Mountain spotted fever had serum antiplatelet antibodies and all 15 dogs infected experimentally with *Rickettsia rickettsii *developed such antibodies by day 26 post infection [67].

The role of the immune system in the pathogenesis of the haemolytic anaemia that characterizes canine *Babesia *infection has been investigated. Studies of *Babesia gibsoni*-infected dogs in Japan demonstrated the presence of IgG and IgM red cell-associated antibodies and the specificity of these antibodies for erythrocyte membrane antigens [73]. A recent European study has suggested that Coombs test positivity is more likely to be associated with *Babesia vogeli *infection rather than that caused by *Babesia canis *[74], although an earlier study from North America reported Coombs positivity in 25/28 cases of *B. gibsoni *infection and 6/9 cases of *B. canis *infection [75]. In contrast, canine haemoplasma infections do not appear to act as a trigger for immune-mediated haemolytic anaemia in this species [76]. The thrombocytopenia that occurs in canine babesiosis may also in part have an immune-mediated pathogenesis and platelet-bound antibodies have been demonstrated in *B. gibsoni*-infected dogs [77].

Dogs infected experimentally with *Bartonella vinsonii *subsp. *berkoffii *developed mild cyclical or sustained immunosuppression related to a reduction in circulating CD8^+ ^T lymphocytes, but had elevation of CD4^+ ^T cells and reduced B cell expression of MHC class II within lymph nodes [78,79]; however, there remains little known of the immune response to *Bartonella *in the dog.

A recent study of Bernese Mountain dogs, a breed predisposed to borreliosis and the proposed associated glomerulopathy; has shown that deficiency of the third component of complement does not appear to underlie either predisposition [80]. The immune response occurring within the synoviae of dogs with borreliosis has not yet been well-characterized, but IL-8 expression has been defined [81].

## Vaccination for canine vector-borne diseases

This section summarizes the current commercially-produced vaccines designed to limit these infections in the dog, but does not generally review the numerous developmental studies of vaccine candidates. Perhaps the most widely available are the *Borrelia *vaccines that are sold in North America and Europe [82]. These are either adjuvanted whole cell lysates (e.g. Merilyme, Merial; LymeVax, Fort Dodge) or vaccines containing a recombinant version of the outer surface protein (Osp) A of the organism that are adjuvanted (e.g. ProLyme, Intervet-Schering Plough) or non-adjuvanted (e.g. Recombitek Lyme, Merial). Most whole cell bacterins are based on *Borrelia burgdorferi *sensu stricto, but one European vaccine includes *Borrelia garinii *and *Borrelia afzelii *which are more relevant species in that area (Biocan B, Bioveta). The major serological response to the whole cell vaccines is to the OspA and OspC proteins. The OspA antibodies are bactericidal and prevent re-infection by killing the spirochaetes in the gut of the fed tick via a complement-dependent mechanism. However, these vaccines fail to protect 20-40% of vaccinated dogs from *Borrelia *infection, which is suggested to reflect the possibilities that: (1) the organism down-regulates OspA expression when a tick commences feeding, (2) the borreliacidal anti-OspA antibodies may be genospecies specific, or (3) that the tick may carry an OspA-negative *Borrelia *[83].

Recombinant OspC vaccines are not licensed, but have been shown experimentally to inhibit colonization of the tick salivary gland, thereby blocking transmission to the vertebrate host. OspC is expressed both in the tick midgut and salivary gland and in the early stages of infection in the host. A bivalent *B. burgdorferi *bacterin (Nobivac Lyme, Intervet-Schering Plough) containing isolates that engender both OspA and OspC antibodies has recently been tested in dogs challenged 1 year post vaccination with *Borrelia*-infected ticks. Compared with unvaccinated controls, vaccinates remained free of joint infection, lameness and synovitis, did not seroconvert to infection-specific antigens (determined by western blotting and use of the Idexx SNAP 4Dx^® ^test kit) and cleared *Borrelia *organisms from the skin adjacent to tick attachment sites by 2 months post exposure [83].

The major consideration related to these non-core *Borrelia *vaccines is that of when their administration might be appropriate; in a situation where exposure (seropositivity) is far more common than clinically significant disease [84]. The decision to use such products in an individual dog should be based upon knowledge of the geographical distribution of the causative organism, the particular species of *Borrelia *present in that region and whether there is vaccinal cross-protection, and the lifestyle and exposure risk (e.g. exercise in wooded areas that are home to *Ixodes *ticks) of that animal [85]. The products all have 1 year licensed duration of immunity and should not be considered an alternative to rigorous control of exposure to ectoparasites.

In Europe, two commercially produced vaccines against canine babesiosis are available. The first is a saponin-adjuvanted vaccine containing soluble parasite antigens (SPA) produced by *B. canis *(Pirodog, Merial) with a licensed duration of immunity of 1 year. The vaccine is unlikely to cross-protect against other *Babesia *species. A saponin adjuvanted bivalent vaccine containing SPA produced by *B. canis *and *Borrelia rossi *(Nobivac Piro, Intervet-Schering Plough) has been shown to provide protection against homologous and heterologous challenge. Vaccinated beagle dogs challenged with *B. rossi *shortly after the last of a series of vaccines had reduction in clinical signs, parasitaemia and plasma concentrations of SPA compared with unvaccinated controls [86]. In contrast, vaccinated dogs challenged with *B. canis *have reduction in clinical signs and plasma SPA, but not parasitaemia [87]. This suggests that the vaccine may have different protective effects - inhibiting infection and disease in the case of *B. rossi *challenge, but inhibiting severity of clinical disease without reducing parasitaemia following challenge with *B. canis*. The latter may occur if the vaccine-induced antibodies were able to prevent the initial vasodilatory shock that leads to microvascular agglutination and hypercoagulation in infected dogs. This pathogenesis is proposed to underlie *B. canis*, but not *B. rossi *infection [88,89]. The vaccine has a licensed duration of immunity of 6 months and is also available in South Africa. There are few available field efficacy data for these vaccines and any risk-benefit analysis for an individual dog should consider the necessity for biannual administration of an adjuvanted non-core vaccine. Again, tick control is of much greater importance than administration of a vaccine for this disease.

The most interesting vaccines in this group are those licensed for the protection of dogs from *Leishmania *infection in Brazil [90]. Although numerous vaccine candidates have been tested over many years only two products are currently available commercially [91]. There have been numerous published studies evaluating the saponin-adjuvanted vaccine incorporating the fucose mannose ligand (FML) enriched for the surface glycoprotein 63 of the parasite (Leishmune^®^, Fort Dodge Animal Health). This product induces protective immunity with IgG seroconversion and development of a robust response to intradermal administration of *Leishmania *lysates. Vaccinated dogs also have a specific *Leishmania*-specific IgG subclass profile [92]. Discrimination between vaccinated and infected dogs is important in a country where serological screening and culling of dogs is undertaken by the Ministry of Health [93]. It has been suggested that the potential for misidentification of vaccinates has led to reluctance of veterinarians in Brazil to offer the vaccine, but in reality it appears that the current serodiagnostic test rarely detects vaccinates [94]. After vaccination, cultures of blood lymphocytes stimulated with *Leishmania *antigen or FML have higher expression of IFN-γ and a reduced proportion of CD4^+^CD25^+ ^putative Treg cells [95]. Similarly, there are more CD4^+ ^T cells in the blood of vaccinated dogs that co-express IFN-γ protein as determined by dual-colour flow cytometry [96]. There is excellent reported clinical efficacy and duration of immunity of 1 year. One large trial was conducted of 550 vaccinates and 588 unvaccinated controls subjected to natural challenge by living in a *Leishmania *endemic area. Two years post vaccination, only 1% of vaccinates died from leishmaniosis (compared with 39% of controls) and only 1.2% of the vaccinates developed clinical signs of the disease (compared with 20.6% of controls) [97]. Most excitingly, where the product has been used it has had an impact on the prevalence of both canine and human infection in the same area, presumptively due to the effect of the vaccine in blocking transmission of the parasite [94].

When enriched with a double dose of saponin adjuvant, the vaccine has also been used successfully as an adjunct immunotherapeutic agent in dogs being treated medically with allopurinol or allopurinol in combination with amphoteracin B. The effect of combined medical and immunotherapy was to reduce clinical and parasitological signs 3 months after initiating therapy and at 8 months to significantly reduce the proportion of treated dogs that remained PCR-positive [98]. A second vaccine, under development for human use, has also been evaluated for immunotherapeutic purposes in affected dogs. The multicomponent protein vaccine Leish-111f formulated with monophosphoryl lipid A in stable emulsion (MPL-SE) had greater therapeutic efficacy in dogs with less severe clinical disease, but treatment with the MPL-SE adjuvant alone also led to clinical benefit [99].

The second canine *Leishmania *vaccine available in Brazil is Leishtec^® ^(Hertape Calier Saúde Animal SA), which is a saponin-adjuvanted vaccine containing a recombinant version of the amastigote-specific A2 antigen of *L. donovani*. The vaccine has a 1-year duration of immunity [100]. Beagle dogs receiving this vaccine seroconvert to the A2 protein but not to entire *Leishmania *promastigote extract, enabling distinction between vaccinated and infected dogs. Cultured blood lymphocytes from vaccinated dogs express elevated levels of IFN-γ, but not IL-10 protein as detected by capture immunoassay. Fewer vaccinated dogs (28.5% of 7 dogs) developed clinical signs post experimental challenge than unvaccinated dogs (71/5% of 7 dogs), and in vaccinates these signs developed later (12 months) than in the unvaccinated controls (3-6 months) [100].

The first European canine *Leishmania *vaccine is due for release in 2011. This product (CaniLeish^®^; Virbac S.A.) consists of excreted secreted proteins (ESP) from *Leishmania infantum *- the dominant antigen of which is the promastigote surface antigen (PSA). The vaccine is indicated for the active immunization of *Leishmania*-negative dogs from 6 months of age and claims to reduce the risk of developing active infection and clinical disease. The product has a 1-year duration of immunity. Other European investigations have examined the *Li*ESAp-MDP vaccine, which contains the 54 kDa excreted protein of *L. infantum *adjuvanted with muramyl dipeptide. In a field trial of spontaneous infection in and endemic area of France, the incidence of infection in 165 vaccinated dogs (2 years post vaccination) was 0.6% versus 6.9% in 175 unvaccinated controls [101].

Although these *Leishmania *vaccines are adjuvanted and administered annually, as non-core vaccines there is much to recommend their use in endemic areas of disease with a high rate of infection in the human population. There are excellent field data to support their efficacy and serological discrimination between vaccinated and infected animals appears feasible. Again, vaccination should be regarded as one of multiple strategies (including control of sandfly exposure and control of stray dogs) in the management of this disease within the population.

Experimental studies have also addressed the possibility of producing vaccines against arthropods for the dog, but these are far from a commercial reality. For example, naïve dogs vaccinated with extracts of salivary gland or midgut from *R. sanguineus *and challenged experimentally 7 and 21 days post vaccination, showed reduced tick attachment (for both vaccine extracts), feeding period and engorgement weight (for salivary vaccine) and fecundity (for midgut vaccine). In the same study, a control group of dogs repeatedly exposed to *R. sanguineus *also showed transient reductions in these parameters, suggesting development of a degree of spontaneous immunity to the tick [102].

## Conclusions

It is clear that our understanding of the pathology and immunology of the CVBDs lags behind the recent rapid advances made in the molecular characterization of the causative organisms, their diagnosis and the epidemiology of infection. The tools to accomplish such studies are now generally available and the development of new vaccines and immunotherapeutics will require their application to expand our fundamental knowledge base.

## Competing interests

Publication of the CVBD6 thematic series has been sponsored by Bayer Animal Health GmbH. The author is a member of the Bayer CVBD World Forum.
